# Heat Shock Protein 60 Is Involved in Viral Replication Complex Formation and Facilitates Foot and Mouth Virus Replication by Stabilizing Viral Nonstructural Proteins 3A and 2C

**DOI:** 10.1128/mbio.01434-22

**Published:** 2022-09-15

**Authors:** Jianli Tang, Sahibzada Waheed Abdullah, Pinghua Li, Jin'en Wu, Chenchen Pei, Suyu Mu, Yunlu Wang, Shiqi Sun, Huichen Guo

**Affiliations:** a State Key Laboratory of Veterinary Etiological Biology, National Foot-and-Mouth Disease Reference Laboratory, Lanzhou Veterinary Research Institute, Chinese Academy of Agricultural Sciences, Lanzhou, Gansu, China; b College of Animal Science, Yangtze University, Jingzhou, Hubei, China; c College of Animal Science, University of Tibet Agricultural and Animal Husbandry, Linzhi, Tibet, China; University of Calgary

**Keywords:** HSP60, FMDV, 3A, 2C, replication complex

## Abstract

The maintenance of viral protein homeostasis depends on the machinery of the infected host cells, giving us an insight into the interplay between host and virus. Accumulating evidence suggests that heat shock protein 60 (HSP60), as one molecular chaperone, is involved in regulating virus infection. However, the role of HSP60 during foot-and-mouth disease virus (FMDV) replication and its specific mechanisms have not been reported. We demonstrate that HSP60 modulates the FMDV life cycle. HSP60 plays a role at the postentry stage of the viral life cycle, including RNA replication and mRNA translation; however, HSP60 does not affect viral replication of Seneca Valley virus (SVA) or encephalomyocarditis virus (EMCV). We found that HSP60 is involved in FMDV replication complex (RC) formation. Furthermore, our results indicate that HSP60 interacts with FMDV nonstructural proteins 3A and 2C, key elements of the viral replication complex. We also show that HSP60 regulates the stability of 3A and 2C via caspase-dependent and autophagy-lysosome-dependent degradation, thereby promoting FMDV RNA synthesis and mRNA translation mediated by the RC. Additionally, we determined that the apical domain of HSP60 is responsible for interacting with 3A and 2C. The N terminus of 3A and ATPase domain of 2C are involved in binding to HSP60. Importantly, HSP60 depletion potently reduced FMDV pathogenicity in infected mice. Altogether, this study demonstrates a specific role of HSP60 in promoting FMDV replication. Furthermore, targeting host HSP60 will help us design the FMDV-specific antiviral drugs.

## INTRODUCTION

Foot-and-mouth disease virus (FMDV), the causative agent of foot-and-mouth disease (FMD), is a small nonenveloped RNA virus belonging to the genus *Aphthovirus* of the family *Picornaviridae* (1, 2). FMD, a highly contagious and economically important disease of cloven-hoofed animals, remains a threat to the livestock industry (1).

The FMDV genome, a positive-sense RNA of approximately 8.4 kb, contains a single open reading frame that encodes a polyprotein. Following receptor-mediated entry, FMDV RNA is delivered into the cytoplasm, where the viral RNA is translated to the polyprotein, which is further processed into three capsid proteins (VP0, VP1, and VP3) and eight nonstructural proteins (L, 2A, 2B, 2C, 3A, 3B, 3C, and 3D) by viral protease (3). Notably, FMDV infection causes the shutdown of cap-dependent translation and initiates the 5′-end-independent mechanism driven by internal ribosome entry site (IRES) elements (4, 5). Viral RNA replication is coupled with mRNA translation in the virus life cycle. First, FMDV genomic RNA mediates the synthesis of a negative-strand RNA to form a double-stranded RNA (dsRNA). Next, the minus-strand RNA serves as a template for synthesizing positive RNA, which is packaged into capsids or translated into viral proteins (4). The capsid assembly is a crucial step required for the production of the FMDV, which starts with the assembly of VP0, VP1, and VP3 to form the basic assembly subunit known as the protomer (5S). Five protomers subsequently assemble into pentamers (12S). Further assembly of 12 pentamers yields the 75S empty capsid. Finally, progeny RNA assembles into virions via encapsidation; then, VP0 undergoes a maturation cleavage to produce VP2 and VP4 (1, 5), and infectious virus is formed (146S). However, whether the progeny RNA is inserted into the empty capsid or the pentamers remains unclear (6, 7).

Like many RNA viruses, FMDV infection causes the rearrangement of intracellular membranes and induces the formation of membrane structures, termed replication complexes (RC), where virus replication takes place, including FMDV RNA replication and mRNA translation (8, 9). In addition, the RC forms in a highly controlled process requiring multiple host proteins and viral nonstructural proteins, such as FMDV 3A and 2C (10, 11). FMDV 3A, the most divergent membrane-binding viral protein in picornaviruses, plays an essential role in RC formation and is also involved in virulence and determining host range (12–14). FMDV 3A contains an N-terminal domain (residues 1 to 60) and a C-terminal domain (residues 77 to 153) that are separated by a hydrophobic transmembrane domain (HD, residues 61 to 76). The highly conserved N terminus and HD domain are thought to be involved in FMDV replication and interaction with the host cellular membrane or proteins (15, 16). In contrast, the C terminus of FMDV 3A is highly variable, and its function has not been completely elucidated, although it may play a role in determining the host range (14).

Another crucial component of the FMDV RC is viral nonstructural protein 2C, the complex and largest viral protein anchored to the cell membrane (17, 18). The highly conserved 318-amino-acid (aa) polypeptide contains an amphipathic helix in its N terminus (residues 17 to 34) and is considered necessary for membrane rearrangement and RC formation. The ATPase domain (aa 60 to 270) lies downstream of the helix, formed by three motifs, and exhibits ATPase activity. The C terminus is conserved, but its function is still unknown (19–21). In addition, previous studies have shown that 2C participates in the life cycle of picornavirus, including virus uncoating, viral RNA binding, and RNA encapsidation (20, 22). Although many studies have indicated the significance of virus-host interaction in regulating FMDV replication, the interacting partners and the details of the mechanisms by which 3A or 2C functions in the FMDV life cycle have not yet been fully elucidated.

Heat shock proteins (HSPs), also known as molecular chaperones, are highly conserved and abundantly distributed in the cellular environment, which can be stimulated by various undesirable factors, including pathogen infection, heat stress, and inflammation. Under stress conditions, HSPs act as a defense mechanism to protect the cell by regulating multiple physiological processes, such as apoptosis, autophagy, protein folding, and immune responses (23, 24). Accumulating evidence implies that HSPs are involved in modulating infection of many viruses. For example, inhibition of HSP70 with a specific inhibitor abrogated Kaposi's sarcoma-associated herpesvirus (KSHV) RC formation and blocked viral RNA polymerase II from properly binding to the viral genome promoter (25). In addition, HSP70 suppression also inhibited viral replication of members of the *Flaviviridae*, including dengue virus and Zika virus (26, 27). Similarly, HSP90 is required for bluetongue virus propagation by inhibiting the degradation of viral protein by blocking the proteasome pathway (28). HSP90 mediates P1 folding during poliovirus infection to achieve the correct conformation and prevent proteasomal-dependent degradation (29).

HSP60 acts as a molecular chaperone in cooperation with HSP10, which is mainly responsible for interacting with target polypeptides in an ATP-dependent manner and exploits its ATPase activity to assist substrate protein folding properly (30, 31). Several studies showed that HSP60 was involved in regulating virus infection. For example, HSP60 precluded porcine reproductive and respiratory syndrome virus replication by positively regulating interferon production in a mitochondrial antiviral signaling protein (MAVS)-dependent manner (32). HSP60 interacted with hepatitis B virus (HBV) polymerase, which participates in protein priming and packaging its genome into capsids, facilitating HBV replication (33). Depleting HSP60 attenuated NLRP3-mediated inflammation during Japanese encephalitis virus (JEV) infection, leading to decreased inflammation and increased survival of JEV-infected mice (34). Moreover, significantly increased expression of HSP60 was observed in dengue virus-infected U937 cells, and downregulation of HSP60 resulted in a decrease in intracellular viral load; however, the details remain elusive (35). Recent research showed that the inhibition of HSP90 impeded P1-2A (FMDV precursor protein) processing and further suppressed pentamer formation (36). However, it remains unclear whether HSP60 orchestrates FMDV replication.

We report that both HSP60 and its cofactor HSP10 are essential for FMDV replication. HSP60 affects viral RNA synthesis and mRNA translation by promoting the stability of 3A and 2C, elements of the RC, without being involved in virus entry or viral precursor folding or processing, and the stability of capsid proteins. HSP60 plays a key role in FMDV RC formation. We also demonstrate that HSP60 depletion does not affect Seneca Valley virus (SVA) or encephalomyocarditis virus (EMCV) replication. *In vivo* experiment was also carried out to evaluate the infectivity of FMDV in an HSP60 knockdown mouse.

## RESULTS

### HSP60 is a specific host factor for viral replication of FMDV.

**FIG 1:**
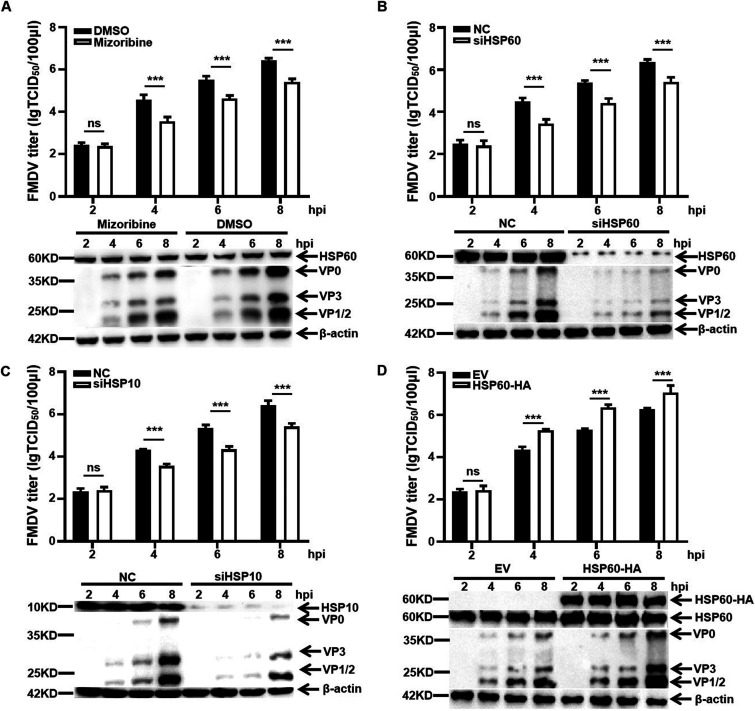
HSP60 and HSP10 are crucial for FMDV replication in PK-15 cells. (A) PK-15 cells were treated with mizoribine at 100 μM for 2 h and then infected with FMDV type O at an MOI of 1. The samples were collected at different time points and were subjected to TCID_50_ assay and Western blotting. (B) PK-15 cells were transfected with siRNA targeting HSP60 (siHSP60) or negative control (NC). At 36 h after transfection, PK-15 cells were infected with FMDV O at an MOI of 1. The FMDV protein level and titer were detected. (C) PK-15 cells were transfected with siRNA targeting HSP10 (siHSP10) or negative control (NC). At 36 h after transfection, PK-15 cells were infected with FMDV O at an MOI of 1. The FMDV protein level and titer were detected. (D) PK-15 cells were transfected with an HA-tagged empty plasmid (EV-HA) or fused to full-length HSP60 overexpression plasmid (HSP60-HA) for 24 h. PK-15 cells were infected with FMDV O at an MOI of 1, and Western blotting and titer assays were performed. Data are means and SD of the results of three independent experiments. ***, *P* < 0.001; ns, not significant.

To investigate the role of HSP60 during FMDV infection, the specific HSP60 inhibitor mizoribine was used to form a complex with HSP60, thereby affecting its folding activity (37). PK-15 cells were treated with a non-cytotoxic concentration of 100 μM and assessed by MTT [3-(4,5-dimethyl-2-thiazolyl)-2,5-diphenyl-2H-tetrazolium bromide] assay (see Fig. S1A in the supplemental material). FMDV replication was determined by virus titration assay and Western blotting (WB). The results showed that the inhibition of HSP60 significantly reduced viral protein level and virus production at 4, 6, and 8 h postinfection (hpi) (Fig. 1A). Considering the potential virucidal effect of mizoribine, we incubated 100 μM mizoribine with FMDV for 4 h at room temperature. Next, the mixture was diluted 500-fold to 0.2 μM (a low concentration, which lost inhibitory activity), and cells were infected for 24 h. A 50% tissue culture infective dose (TCID_50_) assay was performed to determine FMDV infectivity. The results indicated that mizoribine had no virucidal effect on FMDV (Fig. S1B). Altogether, our results demonstrated that HSP60 folding activity is crucial for FMDV replication.

10.1128/mbio.01434-22.1 FIG S1 Screening for efficiency siRNA targeting HSP60 and a non-cytotoxic concentration. (A and E to I) Cytotoxic effect of the indicated drugs on PK-15 cells. The PK-15 cells were treated with a series of concentrations of mizoribine (A), GSK-3008348 (E), T-1105 (F), MG132 (G), Z-VAD-FMK (H), and CQ (I) for 24 h. Relative cell viability was determined by MTT assay. (B) Virucidal effect of mizoribine on FMDV. FMDV was incubated with DMSO or mizoribine (100 μM for 4 h), diluted 500-fold, and added to PK-15 cells. FMDV titers were determined. (C) Knockdown efficiency of siRNA targeting HSP60 (siHSP60). PK-15 cells were transfected with three siRNAs targeting HSP60 (siHSP60) or negative control (NC). At 36 h after transfection, PK-15 cells were infected with FMDV O at an MOI of 1. The protein levels of HSP60 and FMDV were detected. (D) The cytotoxic effect of siRNA targeting HSP60 (siHSP60) on PK-15 cells. PK-15 cells were transfected with three siRNAs targeting HSP60 (siHSP60) or negative control (NC) for 36 h, and relative cell viability was determined by MTT assay. Data are means and SD from three independent experiments. ns, not significant; *, *P < *0.05; **, *P < *0.01; ***, *P < *0.001. Download FIG S1, TIF file, 0.7 MB. Copyright © 2022 Tang et al. 2022 Tang et al. https://creativecommons.org/licenses/by/4.0/ This content is distributed under the terms of the Creative Commons Attribution 4.0 International license.

To further explore the role of HSP60 in the FMDV life cycle, overexpression and knockdown assays were performed in PK-15 cells. First, we evaluated the knockdown efficiency of three small interfering RNAs (siRNAs) targeting HSP60, and Western blot results showed that siHSP60 1 is the optimal siRNA (Fig. S1C). Moreover, an MTT assay was performed to verify that siRNA had negligible cytotoxicity for PK-15 cells (Fig. S1D). Next, we selected siHSP60 1 to transfect the PK-15 cells for 36 h. Cells were infected with FMDV at a multiplicity of infection (MOI) of 1 and then harvested for viral protein and titer analysis. HSP60 depletion significantly decreased viral titer at 4, 6, and 8 hpi (Fig. 1B). HSP10, a key cofactor of HSP60, was involved in assisting the folding of substrate protein which HSP60 mediated; however, its functional role in FMDV infection remains unclear. The depletion assay showed that HSP10 knockdown markedly inhibited FMDV replication (Fig. 1C). More significant inhibition of FMDV replication was monitored in cells simultaneously depleted of HSP60 and HSP10 (Fig. S2C). We confirmed our finding by transfecting PK-15 cells with hemagglutinin (HA)-tagged empty vector (EV-HA) or HA-tagged HSP60 overexpression plasmid (HSP60-HA). After transfection for 24 h, cells were infected with FMDV at an MOI of 1. In contrast, FMDV replication was increased dramatically in HSP60 overexpression cells at 4, 6, and 8 hpi (Fig. 1D). Collectively, these results suggested that HSP60 promoted FMDV replication.

10.1128/mbio.01434-22.2 FIG S2 Effect of HSP60 and HSP10 depletion on FMDV, EMCV, and SVA replication. (A and B) BHK-21 and IBRS-2 cells were transfected with siRNA targeting either HSP60 or NC and were infected with EMCV (A) and SVA (B), respectively, at MOI of at 0.5. The knockdown efficiency of HP60 was validated by Western blotting. Next, viral titer was determined with a TCID_50_ assay. (C) PK-15 cells were transfected with siRNAs targeting HSP60 (siHSP60) and HSP10 (siHSP10). At 36 h after transfection, PK-15 cells were infected with FMDV O at an MOI of 1. The FMDV protein level was detected. (D) BHK-21 cells were transfected with siRNAs targeting HSP60 (siHSP60) or/and HSP10 (siHSP10). At 36 h after transfection, BHK-21 cells were infected with EMCV at an MOI of 0.5. The EMCV titer level was detected. (E) IBRS-2 cells were transfected with siRNA targeting HSP60 (siHSP60) or/and HSP10 (siHSP10) for 36 h. IBRS-2 cells were infected with SVA at an MOI of 0.5, and Western blotting and titer assay were performed. Data are means and SD of the results of three independent experiments. ns, not significant. Download FIG S2, TIF file, 0.7 MB. Copyright © 2022 Tang et al. 2022 Tang et al. https://creativecommons.org/licenses/by/4.0/ This content is distributed under the terms of the Creative Commons Attribution 4.0 International license.

To determine whether HSP60 depletion affects the growth of EMCV and SVA, other picornaviruses, HSP60 knockdown experiments were performed in BHK-21 cells and IBRS-2 cells with viral infection, respectively. As shown in Fig. S2A and B, depletion of HSP60 did not affect the replication of EMCV and SVA. The effect of simultaneous HSP60 and HSP10 depletion on EMCV and SVA replication was further detected. As shown in Fig. S2D and E, the simultaneous depletion of HSP60 and HSP10 does not affect SVA or EMCV replication. These results revealed the potential role of HSP60 in developing an antiviral target specific to FMDV but not other picornaviruses.

### HSP60 regulates viral replication of FMDV at the postentry steps.

**FIG 2:**
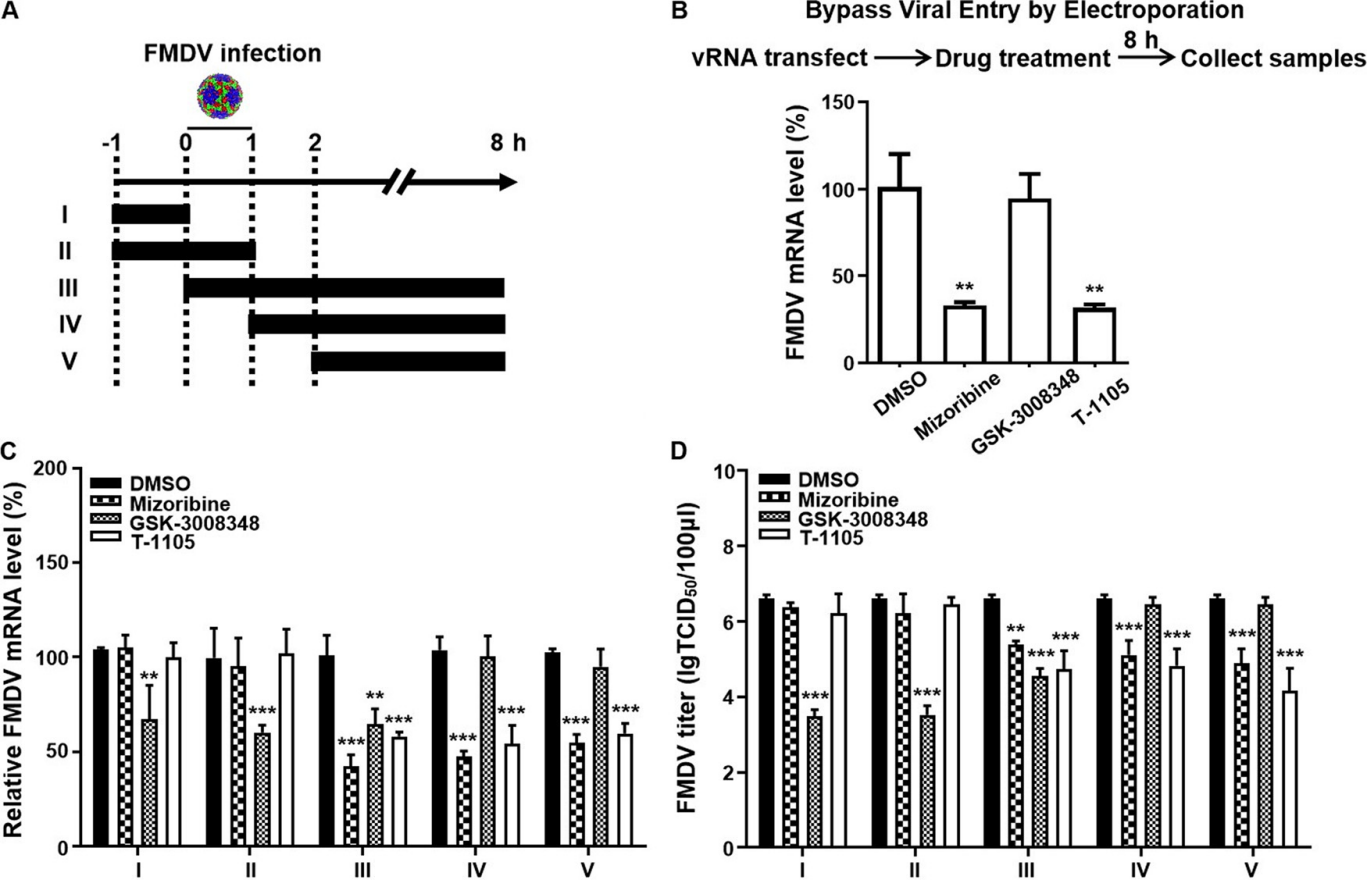
HSP60 facilitates FMDV replication at postentry steps. (A) Schematic diagram of the time course of the drug addition experiment. The HSP60 inhibitor mizoribine, the FMDV entry inhibitor GSK-3008348, and the FMDV polymerase inhibitor T-1105 were used. PK-15 cells were infected with FMDV O at an MOI of 1. FMDV RNA (C) and virus production (D) were measured. (B) Mizoribine treatment suppresses FMDV RNA replication postentry *in vitro*. FMDV RNA was electroporated into PK-15 cells, and inhibitors were added. After 8 h, vRNA was quantified by RT-qPCR. Data are means and SD from three independent experiments. **, *P < *0.01; ***, *P < *0.001.

We performed time-of-drug-addition experiments to clarify which stage of the FMDV life cycle requires HSP60 (Fig. 2A). Moreover, GSK-3008348, an integrin αvβ6 antagonist (38), and T-1105, an FMDV polymerase inhibitor (39), were selected to distinguish the role of HSP60 in entry versus postentry steps. GSK-3008348 suppressed viral RNA (vRNA) replication and virus production when added prior to or concurrently with virus infection (Fig. 2C and D, series I to III). In contrast, T-1105 inhibited FMDV replication only when added concurrently with or after infection (Fig. 2C to D, series III to V). Mizoribine blocked FMDV production and vRNA replication similarly to T-1105, indicating that HSP60 is mainly involved in viral postentry steps (Fig. 2C to D, series III to V). We electroporated *in vitro*-transcribed genomic vRNA directly into cells treated with these drugs to bypass viral entry. GSK-3008348 no longer inhibited FMDV replication, while T-1105 and mizoribine showed strong inhibition of vRNA production (Fig. 2B). In addition, both GSK-3008348 and T-1105 safety concentrations were assessed by MTT assay (Fig. S1E to F). Thus, HSP60 is required for the FMDV life cycle at postentry steps.

### HSP60 is not involved in regulating P1 folding or processing or in the stability of capsid proteins.

**FIG 3:**
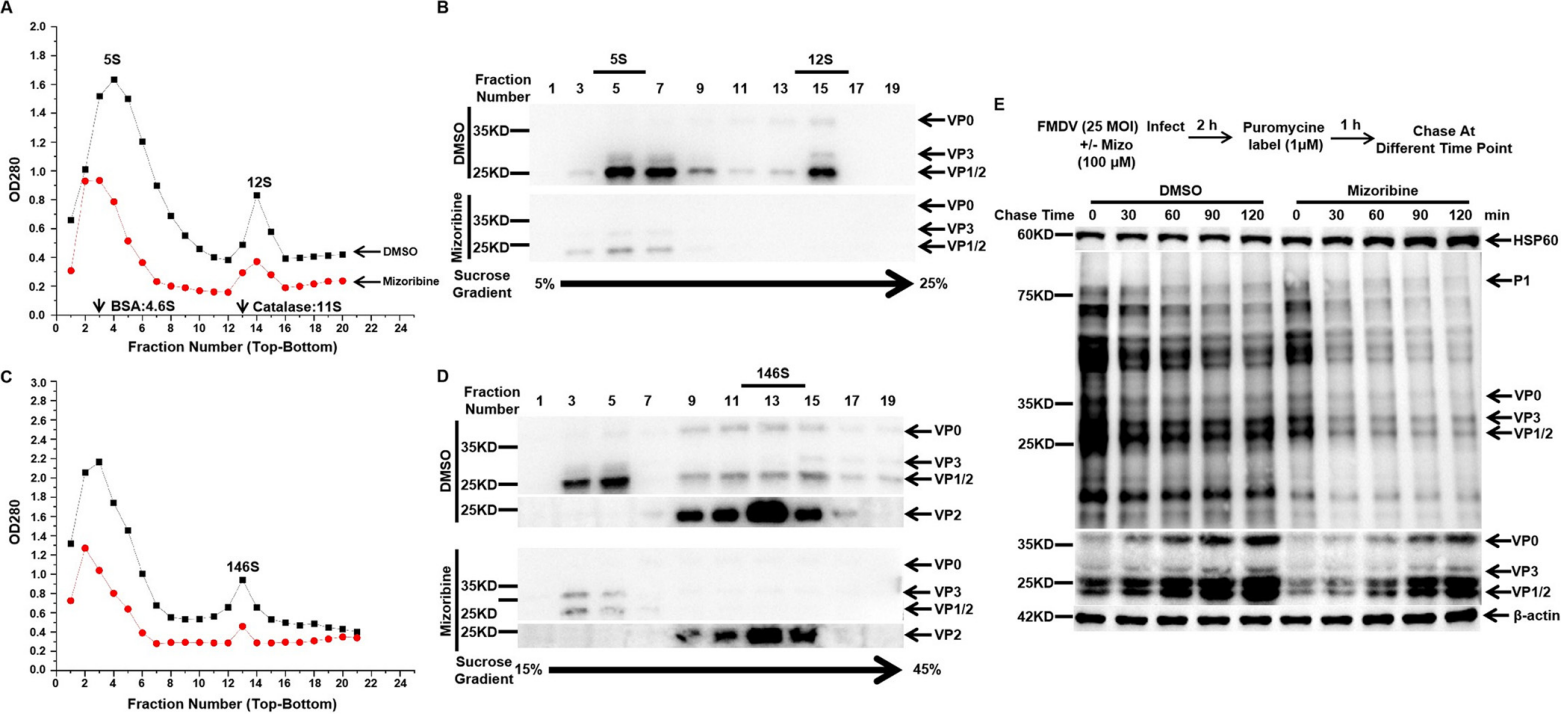
HSP60 is not involved in P1 folding and processing and the stability of capsid proteins. BHK-21 cells were infected with FMDV O at an MOI of 10 with or without mizoribine (100 μM; red dots), and lysates prepared 8 hpi were loaded onto 5-to-25% (wt/vol) (A) or 15-to-45% (wt/vol) (C) sucrose density gradients to separate the protomer (5S) and pentamer (12S) assembly reactions and 146S virion. Next, odd fractions taken from top to bottom were analyzed for 5S protomers and 12S pentamers (B) or 146S virion. (D) by Western blotting using anti-FMDV polyclonal antibody and VP2-specific antibodies. (E) PK-15 cells were infected with FMDV at an MOI of 25. The cells were treated with DMSO or mizoribine at various time points and were pulse-labeled with 1 μM puromycin for an hour before collection. Cell samples were harvested and subjected to Western blot analysis.

Morphogenesis, primarily composed of vRNA synthesis, mRNA translation, and virus assembly, is the least-understood part of the postentry steps of the viral life cycle (5). Previous studies reported that HSP90 facilitated FMDV precursor protein P1-2A processing into capsid proteins and promoted FMDV assembly (36). We speculated that HSP60 might be involved in FMDV assembly. Thus, lysates of BHK-21 cells infected with FMDV for 8 h in the presence or absence of mizoribine were prepared. Then, FMDV was purified as previously described (36, 40, 41). We performed sucrose density gradient (SDG) centrifugation to separate 5S protomers, 12S pentamers (5 to 25% [wt/vol]), and 146S virus particles (15 to 45% [wt/vol]). Moreover, marker proteins (bovine serum albumin [BSA], 4.6S; catalase, 11S) with known sedimentation rates were used to extrapolate unknown products. Every second fraction of the gradient was examined by Western blotting; meanwhile, the absorbance of the fractions was tested at a wavelength of 280 nm. As shown in Fig. 3A, 5S sedimented at the first peak, and the second peak represents 12S. We observed a dramatic decrease in 5S and 12S production in the mizoribine group compared to the dimethyl sulfoxide (DMSO) group. Consistent with the above results, Western blot indicated that FMDV capsid protein production also decreased in the mizoribine group (Fig. 3B). A similar trend was observed in Fig. 3C; 146S virion declined sharply in the mizoribine group. We also used an anti-VP2 monoclonal antibody and FMDV polyclonal antibody to test the 146S virion. Western blot results showed that capsid proteins were dramatically reduced, suggesting that mature-virus production was blocked in mizoribine-treated cells (Fig. 3D). Altogether, these results indicated that the production of virus assembly intermediates, composed of capsid proteins, was suppressed.

Based on the above results, we proposed three conjectures: (i) production of P1, the precursor of capsid proteins, was decreased due to the suppression of vRNA replication or mRNA translation; (ii) the absence of HSP60 activity led to P1 misfolding and processing inhibition, thereby degraded; and (iii) capsid protein stability was decreased. To prove the above hypothesis, we examined the viral protein production with a nonradioactive ribopuromycilation assay (42). As shown in Fig. 3E, all viral proteins, including precursor, structural, and nonstructural proteins, decreased in the mizoribine-treated group. This suggested that the decrease of P1 was mainly caused by the blockage of vRNA replication or mRNA translation. Meanwhile, we tested the interaction between HSP60 and capsid proteins. The results indicated that HSP60 did not interact with VP0, VP1, and VP3 (Fig. S3A). We also transfected capsid proteins into HSP60-deficient PK-15 cells, and the results showed that the stability of capsid proteins was not changed (Fig. S3B). Overall, our results suggested that HSP60 might be involved in viral RNA replication or mRNA translation without affecting viral precursor P1 protein folding or processing or the stability of capsid proteins during FMDV infection.

10.1128/mbio.01434-22.3 FIG S3 HSP60 does not interact with FMDV structural protein and is independent of their stability. (A) Co-IP analysis of endogenous HSP60 and FMDV structural proteins VP0, VP1, and VP3. PK-15 cells were transfected with VP0/VP1/VP3-Flag plasmid, and cell lysates were immunoprecipitated with an HSP60 antibody, followed by immunoblotting with Flag and HSP60 antibodies. (B) HSP60 has no effect on the stability of VP0, VP1, and VP3. PK-15 cells treated with either control siRNA (NC) or HSP60-targeting siRNA (siHSP60) were transfected with VP0/VP1/VP3-Flag plasmid. After 24 h, the relative protein level of VP0, VP1, and VP3 was determined by WB assay. Download FIG S3, TIF file, 0.2 MB. Copyright © 2022 Tang et al. 2022 Tang et al. https://creativecommons.org/licenses/by/4.0/ This content is distributed under the terms of the Creative Commons Attribution 4.0 International license.

### HSP60 mediates FMDV RNA synthesis and mRNA translation.

**FIG 4:**
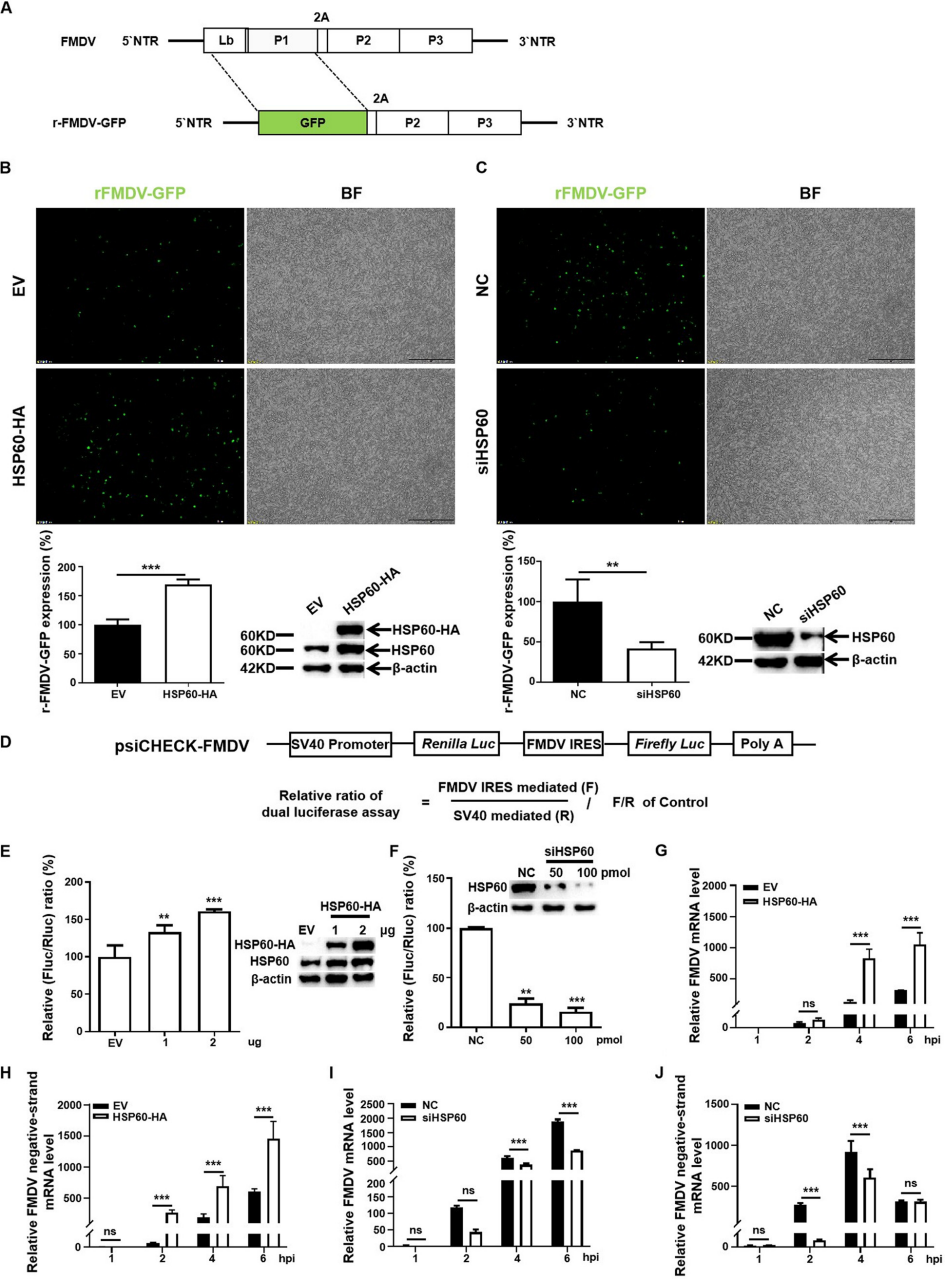
HSP60 positively regulates FMDV RNA replication and translation. (A) Schematic illustration of the replicon rFMDV-GFP construct. (B) BSR-T7 cells were transfected with either the EV or HSP60-HA plasmids. At 12 h posttransfection, cells were transfected with the replicon rFMDV-GFP. After 12 h, the cells were subjected to fluorescence analysis. (C) BSR-T7 cells were knocked down with NC or siHSP60. After 36 h, cells were transfected with replicon rFMDV-GFP. Cells were collected for fluorescence analysis at 12 h posttransfection. (D) Schematic diagram of the bicistronic luciferase construct. (E) PK-15 cells were cotransfected with psiCHECK-FMDV and HSP60-HA. After 24 h, samples were harvested for Western blot analysis and dual-luciferase assay. (F) The knockdown experiment was performed with siHSP60 or NC in PK-15 cells. After 30 h, cells were transfected with psiCHECK-FMDV. Samples were collected at 18 h posttransfection and subjected to immunoblotting assay and luciferase activity analysis. (G and H) PK-15 cells transfected with EV or HSP60-HA were infected with FMDV at an MOI of 1. At indicated time points, total RNA was extracted and subjected to RT-qPCR analysis to determine the levels of total viral RNA (G) and negative-strand FMDV RNA (H). (I and J) PK-15 cells transfected with NC or siHSP60 were infected with FMDV at an MOI of 1. The RNA extraction experiment and RT-qPCR analysis were performed as described in Materials and Methods. Data are the means and SD of the results of three independent experiments. **, *P < *0.01; ***, *P < *0.001; ns, not significant.

We proposed that HSP60 might regulate viral RNA synthesis or mRNA translation based on the above results. Therefore, an FMDV subgenomic replicon with a green fluorescent protein (GFP) reporter gene, rFMDV-GFP, was constructed and transfected into HSP60-deficient or -overexpressing BSR-T7 cells (Fig. 4A). In agreement with the results in Fig. 4B, the levels of specific fluorescence were significantly elevated in cells ectopically expressing HSP60. In contrast, replicon activity was markedly decreased when HSP60 was depleted (Fig. 4C).

During FMDV infection, viral mRNA translation and RNA synthesis take place simultaneously. To explore whether HSP60 is a driving factor for FMDV IRES-driven translation, a bicistronic luciferase plasmid, psiCHECK-FMDV, containing a cap-dependent *Renilla* luciferase gene (*RLuc*) and FMDV IRES-mediated firefly luciferase gene (*FLuc*), was constructed (Fig. 4D). First, PK-15 cells were cotransfected with HSP60-HA and psiCHECK-FMDV, and IRES activity was dramatically increased (Fig. 4E); however, FMDV IRES activity was decreased in HSP60 knockdown cells (Fig. 4F). Overall, our results indicated that HSP60 promoted the FMDV IRES-dependent translation.

To determine the effect of HSP60 on vRNA synthesis, total and negative-strand viral RNA (−RNA) was determined. A significant increase in the level of −RNA at 2, 4, and 6 h posttransfection (hpt) was observed in HSP60-overexpressing cells (Fig. 4G to H). An opposite trend in −RNA level was found when HSP60 was depleted at 2 and 4 hpt (Fig. 4I to J). Our results demonstrated that HSP60 boosted vRNA synthesis. Altogether, these results showed that HSP60 serves as a positive regulator of viral IRES-dependent translation and RNA replication, consequently promoting the efficient replication of FMDV.

### HSP60 affects viral replication complex formation.

**FIG 5:**
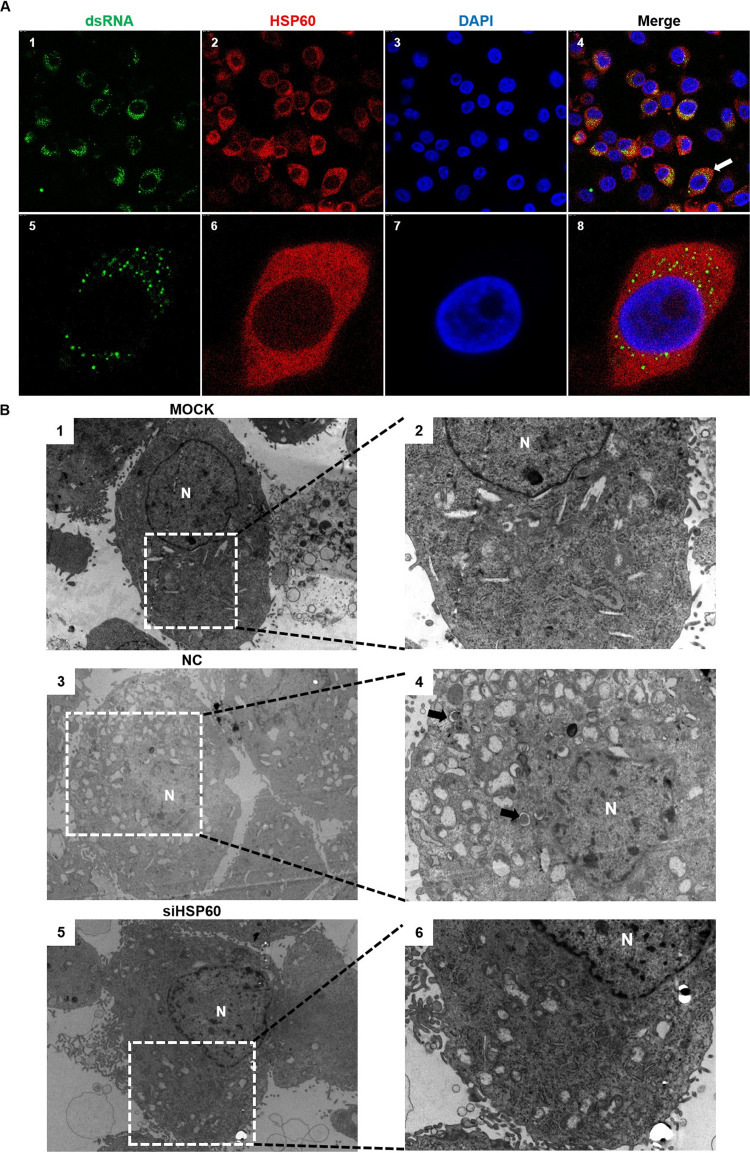
HSP60 is involved in viral replication complex formation. (A) Colocalization of dsRNA and HSP60 in PK-15 cells. Cells were challenged with FMDV O at an MOI of 1 for 3 h, fixed, stained with DAPI (blue) and antibodies against dsRNA (green) and HSP60 (red), and then examined by confocal microscopy. (B) Control and HSP60-depleted cells were mock infected or infected with FMDV at an MOI of 5, and images were obtained by transmission electron microscopy at 3 h postinfection. Virus-induced vesicular clusters (white boxes) and double-membrane vesicles (DMVs) (black arrows) are shown in images 3, 4, 5, and 6. Low-magnification images are shown in images 1, 3, and 5. High-magnification images are shown in images 2, 4, and 6. N, nucleus.

Since viral RNA replication and mRNA translation were significantly decreased in HSP60 depleted cells, we wondered if HSP60 is involved in viral replication complex formation. As a marker for the viral replication complex, double-stranded RNA indicates viral RNA synthesis. Therefore, we confirmed whether HSP60 colocalized with dsRNA to determine whether it is involved in the viral replication complex. The localization of dsRNA and HSP60 was examined in FMDV-infected PK-15 cells. As shown in Fig. 5A, HSP60 shows strong correlation with dsRNA.

Furthermore, transmission electron microscopy (TEM) was performed to observe the RC generation in negative-control and HSP60 knockdown cells. Virus-induced modifications were observed in the perinuclear region (Fig. 5B, images 1, 3, and 5, white boxes). The virus-induced membranous structures were reduced in HSP60 knockdown cells compared to negative controls. Double-membrane vesicles (DMVs) were also observed in negative controls (Fig. 5B, images 2, 4, and 6, black arrows) but not in HSP60-depleted cells. Taken together, the results show that HSP60 is involved in FMDV RC formation.

### HSP60 interacts with nonstructural viral proteins 3A and 2C.

**FIG 6:**
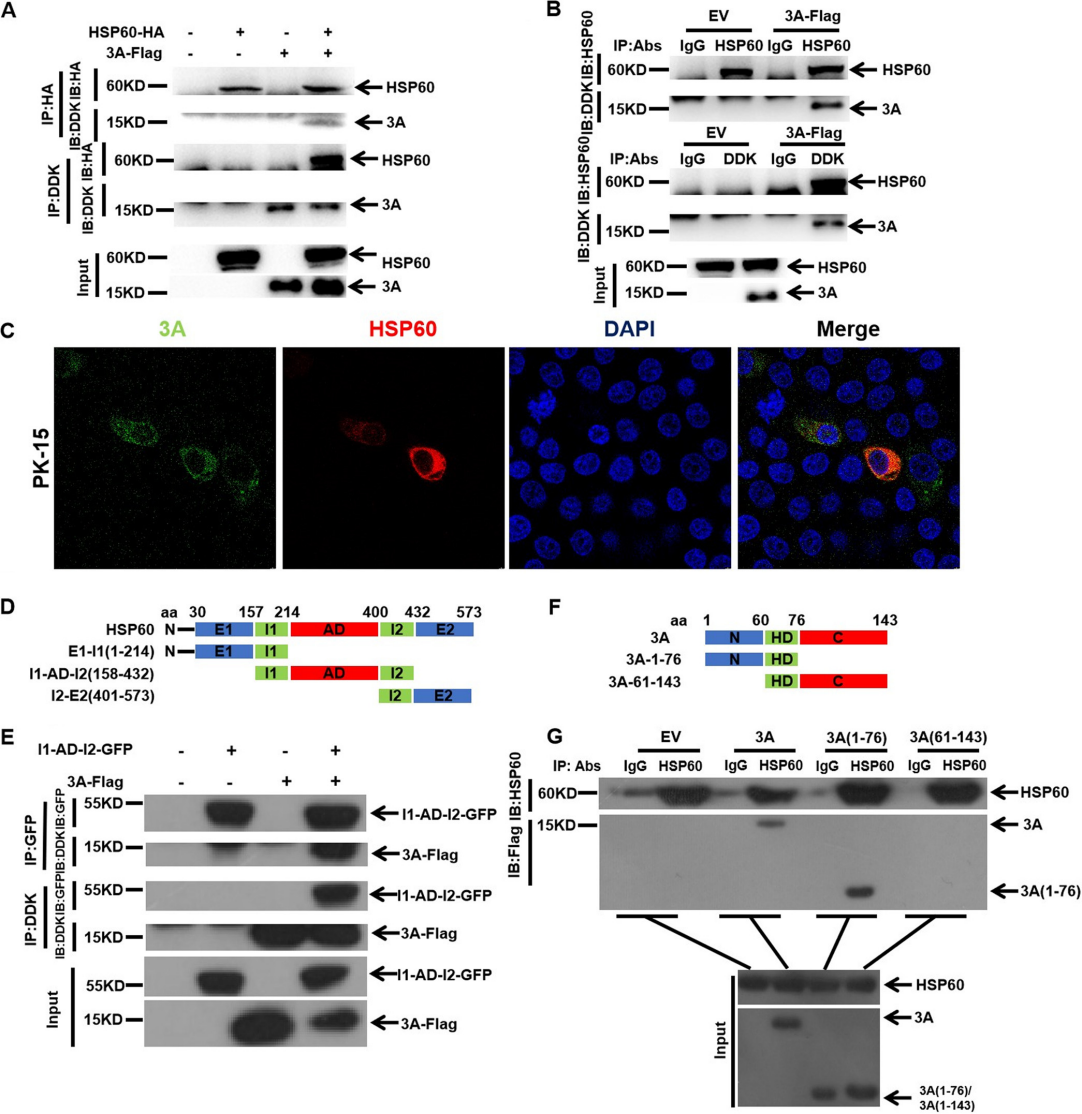
Identifying the interaction between HSP60 and 3A and the functional domain involved in their association. (A) Co-IP analysis of exogenous HSP60 and 3A. PK-15 cells were cotransfected with HSP60-HA and 3A-Flag plasmids, and cell lysates were immunoprecipitated with an HA or DDK antibody, followed by immunoblotting with DDK and HA antibodies. (B) Endogenous HSP60 interacts with 3A. PK-15 cells were transfected with the 3A-Flag plasmid, and a co-IP experiment was carried out as described in Materials and Methods. (C) Colocalization of 3A with HSP60. PK-15 cells were cotransfected with HSP60-HA and 3A-Flag. The immunofluorescence staining experiment was performed with anti-HA (red), anti-DDK (green), and DAPI (blue). (D and F) Schematic representations of individual HSP60 and 3A truncations. (E) Interaction between 3A and I1-AD-I2. PK-15 cells were cotransfected with I1-AD-I2-GFP and 3A-Flag. A co-IP experiment was performed as described in Materials and Methods, and the GFP and DDK antibodies were used. (G) Identification of the domain in 3A responsible for interacting with endogenous HSP60. Truncated 3A mutants were transfected into PK-15 cells, co-IP experiments were performed as described in Materials and Methods, and anti-HSP60 and anti-Flag antibodies were used in the immunoblotting assay.

**FIG 7:**
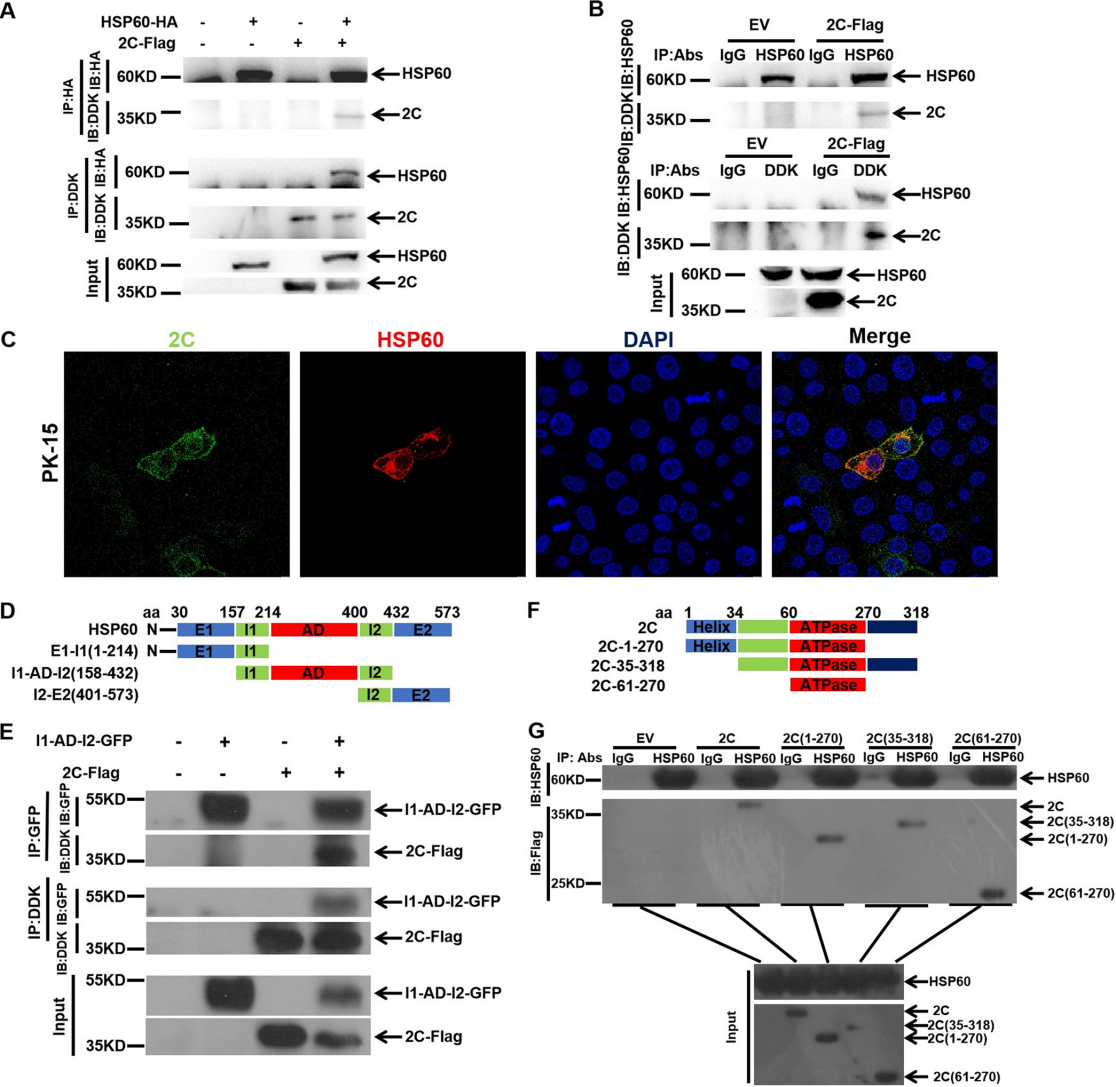
Identifying the interaction between HSP60 and 2C and the functional domain involved in their association. (A) Co-IP analysis of exogenous HSP60 and 2C. PK-15 cells were cotransfected with HSP60-HA and 2C-Flag plasmids, and cell lysates were immunoprecipitated with an HA or DDK antibody, followed by immunoblotting with DDK and HA antibodies. (B) Endogenous HSP60 interacts with 2C. PK-15 cells were transfected with the 2C-Flag plasmid, and a co-IP experiment was carried out as described in Materials and Methods. (C) Colocalization of 2C with HSP60. PK-15 cells were cotransfected with HSP60-HA and 2C-Flag. The immunofluorescence staining experiment was performed with anti-HA (red), anti-DDK (green), and DAPI (blue). (D and F) Schematic representations of individual HSP60 and 2C truncations. (E) Interaction between 2C and I1-AD-I2. PK-15 cells were cotransfected with I1-AD-I2-GFP and 2C-Flag. A co-IP experiment was performed as described in Materials and Methods, and the GFP and DDK antibodies were used. (G) Identification of the domain in 2C responsible for interacting with endogenous HSP60. 2C truncated mutations were transfected into PK-15 cells, co-IP experiments were performed as described in Materials and Methods, and anti-HSP60 and anti-Flag antibodies were used in the immunoblotting assay.

The RC is the central place for vRNA replication and mRNA translation. 3A and 2C, as RC components, might be the targets of HSP60 in regulating FMDV growth. Thus, 3A and 2C expression plasmids (3A-Flag/2C-Flag) and HSP60-HA were cotransfected into PK-15 cells to investigate whether exogenous HSP60 interacts with 3A or 2C. Coimmunoprecipitation (co-IP) and Western blotting showed that exogenous HSP60 interacted with 3A and 2C (Fig. 6A and 7A).

To further investigate whether endogenous HSP60 interacts with 3A or 2C, PK-15 cells were transfected with a 3A-Flag or 2C-Flag. As shown in Fig. 6B and 7B, 3A and 2C interacted with endogenous HSP60. The co-IP results were further confirmed through confocal microscopy, and we found that both 3A and 2C colocalized with HSP60 (Fig. 6C and 7C). These results indicated a strong correlation between HSP60 and 3A or 2C.

HSP60 was composed of three structural domains: the apical domain (AD; ranging from residue 215 to 400), the intermediate domain (ID; residues 158 to 214 and 401 to 432), and the equatorial domain (ED; residues 30 to 157 and 433 to 573). The AD mainly exhibits substrate and HSP10 binding activity, and the ID plays a crucial role in the ligand-induced conformational changes in HSP60. However, the role of the ED is unclear (30, 43). To confirm the domain responsible for binding 3A or 2C, truncated HSP60 mutants were constructed and transfected into PK-15 cells (Fig. 6D and 7D). Our results showed that the AD interacted with 3A and 2C (Fig. 6E and 7E). Meanwhile, we confirmed the functional domains of 3A and 2C, which are important for binding to HSP60. 3A and 2C truncation plasmids (Fig. 6F and 7F) were constructed and transfected into PK-15 cells. The results showed that the 3A N terminus interacted with HSP60 (Fig. 6G). For 2C, its ATPase domain played a key role in binding to HSP60 (Fig. 7G). In summary, we characterized the interaction between HSP60 and 3A or 2C; meanwhile, the functional domains involved were identified.

### Inhibition of HSP60 degrades 3A and 2C through apoptosis and autophagy-lysosome pathways, respectively.

**FIG 8:**
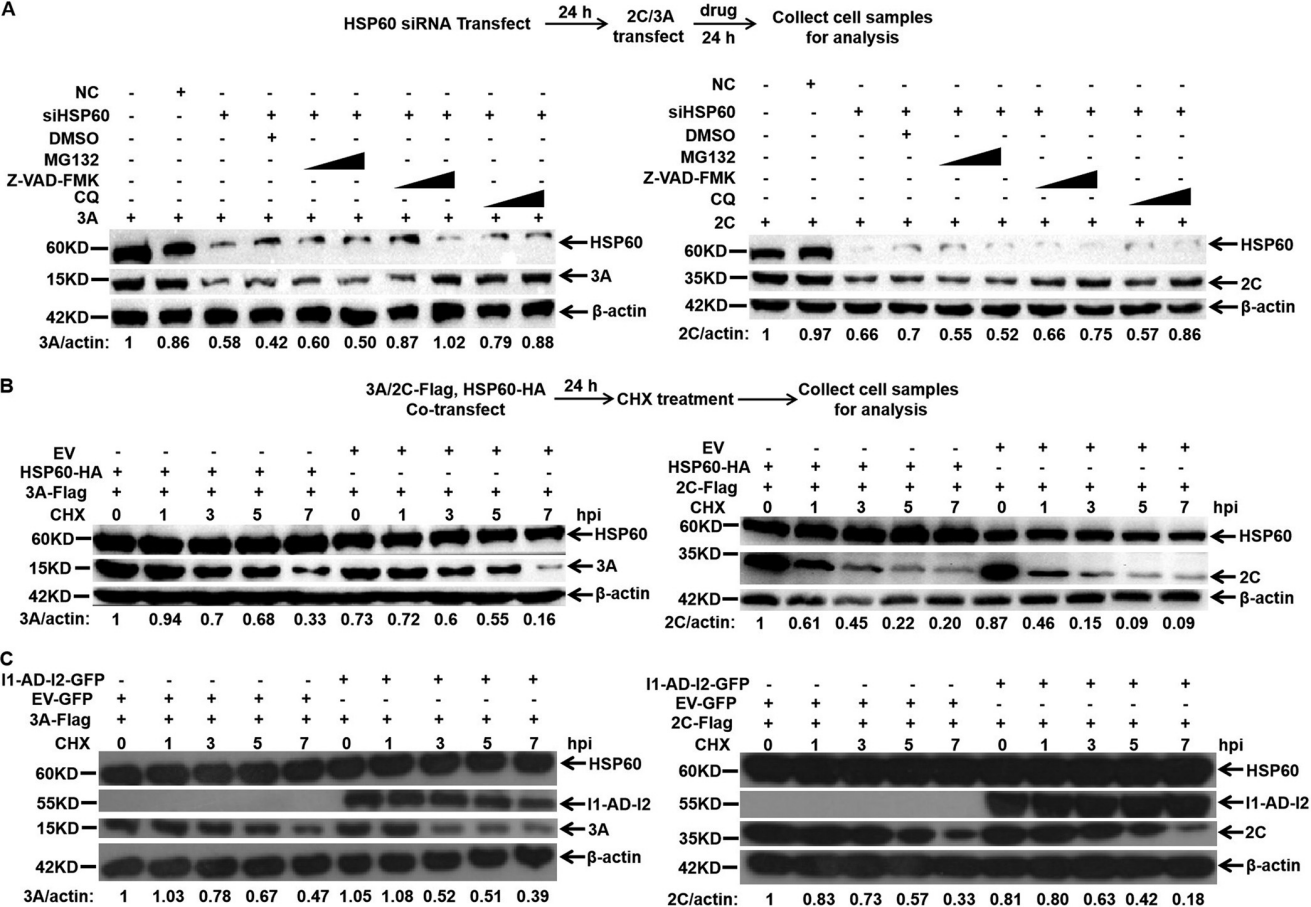
HSP60 regulates the stability of FMDV 3A and 2C proteins in an apoptosis- and autophagy-dependent manner. (A) PK-15 cells treated with either control siRNA (NC) or HSP60-targeting siRNA (siHSP60) were transfected with the 3A-Flag or 2C-Flag plasmid and maintained in the presence of DMSO, MG132 (2 or 20 μM), Z-VAD-FMK (10 or 50 μM), or CQ (20 or 50 μM). After 24 h, the relative protein level of 3A or 2C was determined by WB assay. (B) PK-15 cells were cotransfected with HSP60-HA and the 3A-Flag or 2C-Flag plasmid. After 24 h, the cells were treated with CHX (100 g/mL). Next, the cells were harvested at the indicated times. Immunoblotting was performed to determine the relative abundance of 3A or 2C. (C) PK-15 cells were cotransfected with I1-AD-I2-GFP and 3A-Flag or 2C-Flag plasmids. After 24 h, the cells were treated with CHX (100 g/mL). The expressions of 3A or 2C in collected samples were detected by Western blotting.

HSP60 primarily promotes protein folding and assists the correct conformation, whereas misfolding leads to substrate protein degradation (30, 31). Thus, we hypothesized that HSP60 might modulate the stability of 3A and 2C. To confirm our speculation, HSP60 knockdown PK-15 cells were transfected with 3A-Flag or 2C-Flag plasmid. Consistent with our conjecture, the stability of 3A and 2C was dramatically decreased in HSP60-depleted cells (Fig. 8A). Next, the half-life of 3A and 2C was analyzed by WB in cycloheximide (CHX)-treated PK-15 cells in the presence or absence of HSP60-HA. The results showed that HSP60 overexpression markedly extended the accumulation of 3A and 2C (Fig. 8B). To explore which protein degradation pathway was involved in HSP60 depletion-mediated 3A or 2C degradation, MG132 (a proteasome inhibitor), chloroquine (CQ; a lysosome inhibitor), and Z-VAD-FMK (a general caspase inhibitor) were used. As shown in Fig. 8A, Z-VAD-FMK and CQ can effectively restore protein levels of 3A and 2C, respectively. This suggested that HSP60 sustains the stability of 3A and 2C by regulating apoptosis and autophagy-lysosome pathways. Moreover, the cytotoxicity of the inhibitors was determined by MTT assay (Fig. S1G to I). To explore if the AD domain of HSP60 was responsible for regulating 3A or 2C stability. AD or empty vector and 3A-Flag or 2C-Flag were cotransfected into PK-15 cells in the presence of CHX. Next, 3A and 2C half-life was tested. Intriguingly, the half-life of 3A and 2C in AD overexpressed cells was reduced compared to that in the negative control (Fig. 8C). We guessed that change might be caused by the substrate-binding activity of the AD domain. These data suggested that full-length HSP60, but not the AD, was essential for modulating the degradation of 3A and 2C through apoptosis and autophagy-lysosome pathways.

### Knockdown of HSP60 protects suckling mice from FMDV infection.

**FIG 9:**
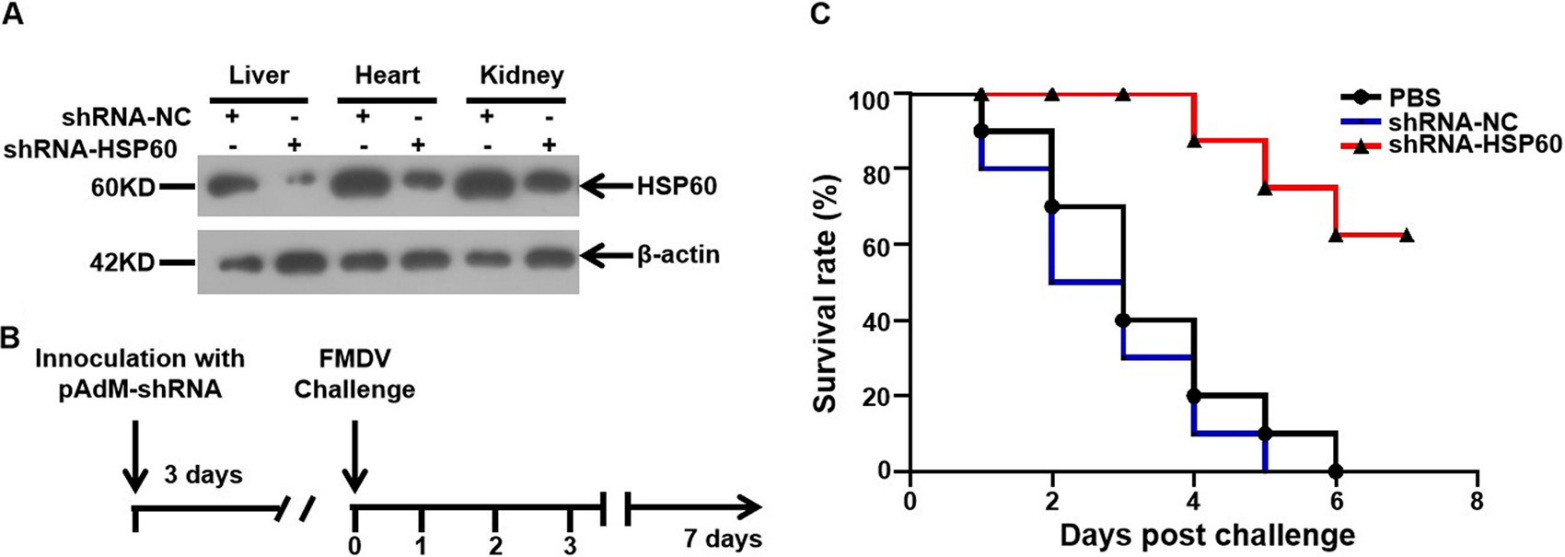
HSP60-depleted suckling mice are less susceptible to FMDV infection. (A) Suckling mice inoculated with pAdM-shNC or pAdM-shHSP60 at 1.0 × 10^8^ PFU. The mice were sacrificed 72 h later, and their livers, hearts, and kidneys were collected and homogenized. Then, the expression level of HSP60 protein in the tissues was determined by Western blotting. (B) Schematic representation of FMDV challenge procedure. Two-day-old suckling mice were inoculated with 1.0 × 10^8^ PFU of pAdM-shNC or pAdM-shHSP60 diluted in 100 μL PBS. After 72 h, the mice were challenged with 20 LD_50_ of FMDV O. (C) Survival rates of FMDV-infected mice treated with PBS, pAdM-shNC, and pAdM-shHSP60. Suckling mice were monitored for up to 7 days.

The potential role of HSP60 as an antiviral target was further investigated in a suckling mouse model of FMDV infection. First, we inoculated suckling mice subcutaneously with pAdM-shHSP60, an adenovirus expressing specific short-hairpin RNA (shRNA) targeting HSP60, for 72 h. Next, the efficiency of HSP60 depletion was assessed. Compared with pAdM-shNC-treated mice, the protein level of HSP60 was decreased in different tissues of the pAdM-shHSP60-treated mice (Fig. 9A). To explore the susceptibility of suckling mice to FMDV infection, 20 median lethal doses (LD_50_) of FMDV were inoculated into suckling mice, and their survival rate was recorded (Fig. 9B). As shown in Fig. 9C, all mice injected with phosphate-buffered saline (PBS) or pAdM-shNC died after 6 days of infection and the 5th day of infection, respectively. Seventy percent of the HSP60-depleted mice survived until the end of the experiment. These results indicated that the knockdown of HSP60 rendered suckling mice less susceptible to FMDV infection.

## DISCUSSION

Due to their limited capacity for replication, picornaviruses depend on the host machinery to accomplish their life cycle, giving us an insight into fundamental cellular processes (43). HSPs, highly conserved cellular proteins found across almost all living organisms, are modulated upon picornavirus infection, which is involved in viral replication (24, 29, 36, 44, 45). For example, EV-A71 exploits different HSP70 isoforms at different stages during its life cycle to replicate (44, 45). HSP90 is required for processing of the FMDV precursor P1-2A to capsid proteins, thus promoting capsid assembly (36). Furthermore, HSP90 is required for proper poliovirus P1 folding and processing (29). The role of HSP60 in picornavirus replication, especially FMDV, remained unclear. Here, we investigated the requirements of HSP60 for FMDV postentry steps and provide evidence of a critical role for HSP60 in FMDV viral RNA replication and mRNA translation. Our results showed that HSP60 depletion resulted in the degradation of FMDV nonstructural protein 3A and 2C, the key components of the FMDV viral replication complex. Importantly, HSP60 depletion also decreased the mortality of FMDV-infected mice and appeared to be a promising antiviral target.

Our results showed that HSP60 and its cofactor HSP10 were essential for FMDV replication (Fig. 1). The life cycle of picornaviruses begins with the binding of the virus to the receptors on the host cell surface. αvβ6, a member of the integrin family, a well-known receptor of FMDV, plays a key role in the FMDV entry step by binding to the RGD motif of VP1 (38). Previous studies showed that HSP60 was distributed on the extracellular surface and might function as the receptor to help pathogen invasion. For example, HSP60 has been identified as the receptor of tumor cell-adapted rotavirus in Reh cells (46). In addition, the Legionella pneumophila adherence to and invasiveness of HeLa cells were inhibited by HSP60-specific antibodies in a dose-dependent manner (47). Thus, we performed time-of-drug-addition experiments to investigate the role of HSP60 in the FMDV entry step (Fig. 2A). Our results showed that HSP60 mainly participated in the postentry steps during FMDV replication but did not affect entry or attachment steps (Fig. 2B to D). After virus internalization, the capsid cleaves and allows the release of viral RNA into the cytoplasm. Next, RNA serves as the template for RNA synthesis and starts IRES-dependent translation of the polyprotein. Morphogenesis, consisting of P1 precursor processing, capsid assembly, RNA encapsidation, and virus maturity, is the most intricate and important part of the life cycle of picornaviruses (5). HSPs have been shown to play a crucial role in virus morphogenesis. During replication of enteroviruses, HSP90 assisted the newly formed poliovirus P1 capsid precursor to attain a properly processing-competent conformation, which could be cleaved into capsid proteins (29). For FMDV, treatment with 17-DMAG [17-(dimethylaminoethylamino)-17-demethoxygeldanamycin], an inhibitor of HSP90, blocked the P1-2A processing into capsid and further inhibited capsid assembly (36).

Moreover, HSP70 was coimmunoprecipitated with the poliovirus (PV) and coxsackievirus (CV) P1 proteins and dramatically extended their half-life. Sucrose density gradient results suggested that HSP70 might be involved in the folding and processing of P1 (48). Based on the above studies, we speculated whether HSP60 is involved in FMDV morphogenesis. Our SDG results showed that suppression of HSP60 activity reduced the production of assembly intermediates (Fig. 3A to D). In addition, ribopuromycilation assay results further suggested that the decreased production of P1 was caused by the inhibition of viral RNA replication or mRNA translation (Fig. 3E). Co-IP results showed that HSP60 did not interact with capsid proteins and had no effect on its stability (Fig. S3A and B). It has been reported that the HSP network regulates virus vRNA synthesis and IRES activity. HSPA1, HSPA8, and HSPA9 were shown to be required for enterovirus A71 (EV-A71) entry and IRES-mediated translation (44). Unlike the above HSP70 isoforms, knockdown of HSPA6 caused the reduction only of luciferase activity driven by the IRES from coxsackievirus A16, echovirus 9, encephalomyocarditis virus, and hepatitis C virus (HCV) (45, 49). In addition, HSP70-1 was upregulated upon coxsackievirus B3 (CVB3) infection, which promoted vRNA replication by stabilizing the CVB3 genome via binding to the AU-rich element located in the 3′ untranslated region of CVB3 genomic RNA (50).

Similarly, HSP90 dysfunction led to L protein, an element of the polymerase of snakehead vesiculovirus, being degraded and further inhibited the transcription and replication of viral RNA (51). Additionally, suppression of HSP90 activity inhibits HCV and influenza virus RNA replication (52, 53). Thus, we attempted to determine the role of HSP60 in FMDV RNA replication and mRNA translation. First, the fluorescence intensity of the FMDV replicon suggested that HSP60 positively regulated vRNA replication or/and mRNA translation (Fig. 4B and C). Next, we determined the luciferase activity and the synthesis of RNA (Fig. 4E to G). As shown in our results, knockdown of HSP60 blocked both vRNA replication and mRNA translation. We confirmed that HSP60 played a positive regulatory role in FMDV genome replication and IRES-mediated translation; however, the precise mechanisms by which HSP60 functions are not fully understood.

Picornavirus infection induces membrane rearrangement, critical for formation of the viral RC, where viral RNA synthesis and mRNA translation occur (8, 9). The RC dysfunction might cause the suppression of the above two steps. Previous studies show that host proteins participate in RC formation (54–56). Here, we demonstrated that HSP60 was required for FMDV RC formation by confocal imaging and electron microscopy (Fig. 5). In addition, the viral structural and nonstructural proteins are involved in the formation of the RC (10, 11). Our results showed that 2C and 3A, the key components of the RC, were clients of HSP60 (Fig. 6A to C and 7A to C). Furthermore, we found that the AD domain of HSP60 plays a key role in interacting with both 2C and 3A (Fig. 6E and 7E). In turn, we identified the key domains of 2C and 3A, which were responsible for interacting with HSP60 (Fig. 6G and 7G). As it is the key cofactor of HSP60, whether HSP10 interacts with 3A or 2C was further investigated. Results show that HSP10 does not interact with 3A or 2C (Fig. S4A and B).

10.1128/mbio.01434-22.4 FIG S4 HSP10 does not interact with FMDV nonstructural protein 3A or 2C. (A) Co-IP analysis of endogenous HSP10 and 3A. PK-15 cells were transfected with the 3A-Flag plasmid, and cell lysates were immunoprecipitated with an HSP10 antibody, followed by immunoblotting with Flag and HSP10 antibodies. (B) Endogenous HSP10 does not interact with 2C. PK-15 cells were transfected with the 2C-Flag plasmid, and a co-IP experiment was carried out as described in Materials and Methods. Download FIG S4, TIF file, 0.1 MB. Copyright © 2022 Tang et al. 2022 Tang et al. https://creativecommons.org/licenses/by/4.0/ This content is distributed under the terms of the Creative Commons Attribution 4.0 International license.

In previous studies, HSPs such as HSP90, and HSP70 were shown to maintain protein homeostasis with their folding-promoting capacity (29, 44, 48, 51). Our results found that HSP60 depletion significantly decreased the stability of viral proteins 2C and 3A. Protein stability is mainly modulated by the ubiquitin-proteasome system, caspase-dependent degradation, or autophagy-lysosome-mediated degradation (57, 58). In the present study, we found that the degradation of 2C and 3A caused by HSP60 depletion was inhibited by Z-VAD-FMK (caspase inhibitor) and CQ (autophagy inhibitor) but not by the proteasome inhibitor MG132 (Fig. 8A). Similarly, HSP60 overexpression significantly extended the half-life of 2C and 3A (Fig. 8B). Altogether, our results suggested that HSP60 was involved in FMDV RC formation by interacting with 2C and 3A and maintained their stability through blocking autophagy-lysosome and apoptosis pathway. Importantly, our results determined how HSP60 is involved in regulating RNA replication and mRNA translation and further modulates FMDV replication.

**FIG 10:**
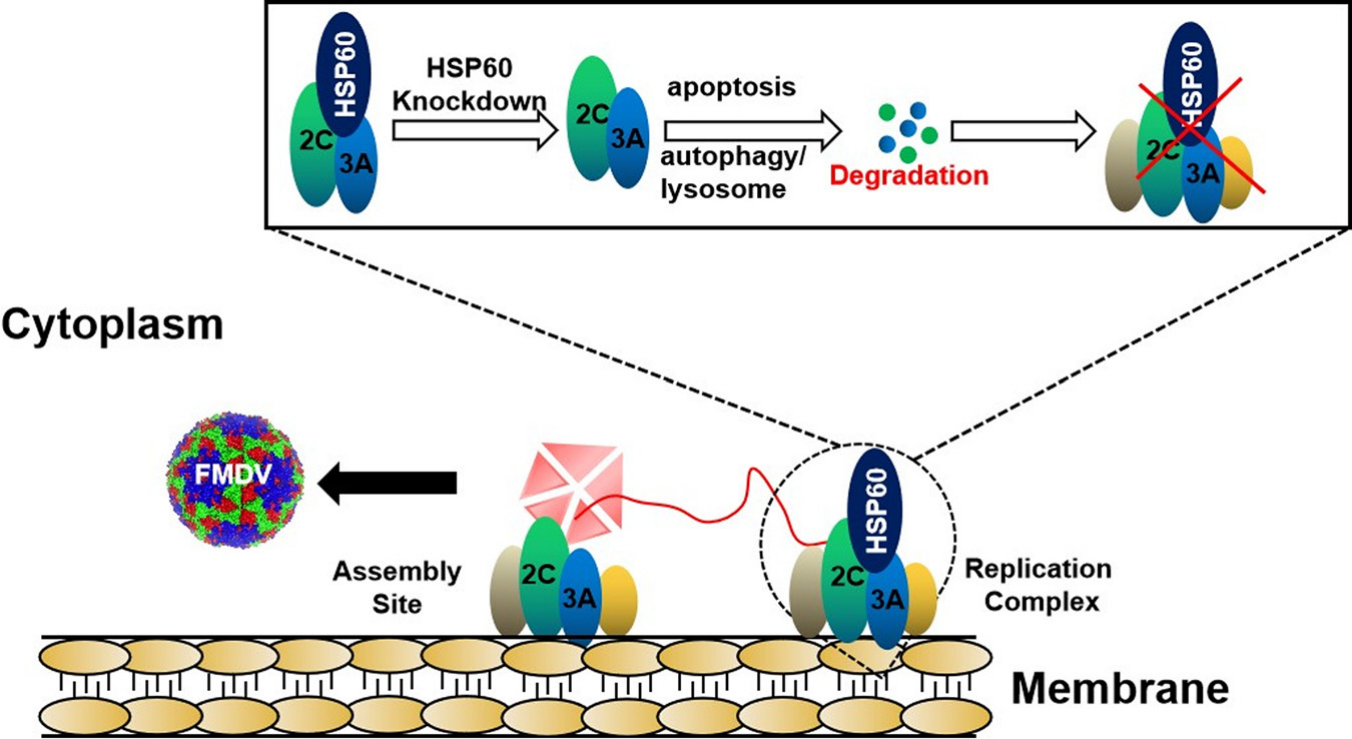
Diagram showing the hypothetical mechanism of HSP60 regulation of FMDV replication. Following FMDV infection, 3A and 2C are synthesized and are involved in viral replication complex formation. HSP60 interacts with 3A and 2C and improves their stability by regulating autophagy and apoptosis. Finally, HSP60 promotes RNA synthesis and mRNA translation by maintaining proper RC function.

In conclusion, our study supports a model whereby HSP60 is involved in FMDV RC formation and promotes RNA replication and mRNA translation by stabilizing nonstructural viral proteins 3A and 2C (Fig. 10). In addition, the animal experiment showed the potential role of HSP60 as an antiviral target (Fig. 9). The findings in this study revealed a novel role for HSP60 in FMDV replication and provided insights that could contribute to the development of therapeutics targeting viral infections.

## MATERIALS AND METHODS

### Ethics statement.

The animal experiments were performed at the biosafety level 3 laboratory of Lanzhou Veterinary Research Institute, Chinese Academy of Agricultural Sciences (permission number SYXK-GAN-2018-0005). According to the Animal Ethics Procedures and Guidelines of the Ministry of Science and Technology of the People's Republic of China, all animal experiments were performed strictly following good animal practices. The study was approved by the Animal Ethics Committee of Lanzhou Veterinary Research Institute, Chinese Academy of Agricultural Sciences (no. LVRIAEC2018-005).

### Cells and viruses.

BHK-21 (baby hamster kidney; ATCC CCL-10), PK-15 (porcine kidney; ATCC CCL-33), and IBRS-2 (swine kidney; Procell CL-0123) cells were maintained in Dulbecco's modified Eagle's medium (DMEM) (Gibco, California, USA) supplemented with 10% fetal bovine serum (FBS) (Gibco), penicillin (100 U/mL), and streptomycin (100 mg/mL) (Gibco) at 37°C under 5% CO_2_. BSR-T7 cells (a kind gift from Karl-Klaus Conzelmann), which stably express T7 RNA polymerase, were grown in DMEM supplemented with 10% FBS, 100 U/mL penicillin, 100 mg/mL streptomycin, and 1 mg/mL G418 (Life Technologies, CA, USA).

FMDV serotype O strain O/China/99 (GenBank accession no. AF506822.2) was maintained by the OIE/National Foot-and-Mouth Disease Reference Laboratory (Lanzhou, China). FMDV was propagated in BHK-21 cells, and the viral titers were determined with a 50% tissue culture infective dose (TCID_50_) assay in BHK-21 cells. SVA (accession no. MN922286), propagated in IBRS-2 cells, and EMCV (accession no. KJ524643), propagated in BHK-21 cells, are stored in our laboratory.

### Antibodies and reagents.

Mouse anti-HSP60, anti-DDK monoclonal antibody, and rabbit anti-HSP60 monoclonal antibody were purchased from Proteintech (Wuhan, China). Mouse anti-HA monoclonal antibody was purchased from Thermo Fisher Scientific (Waltham, MA, USA). Mouse antiactin monoclonal antibody was purchased from CWBIO (Beijing, China). Rabbit anti-GFP monoclonal antibody was purchased from Abcam (Cambridge, MA, USA). Rabbit anti-HSP10 and anti-Flag monoclonal antibodies and the secondary antibodies conjugated with horseradish peroxidase (HRP), fluorescein isothiocyanate (FITC), and tetramethyl rhodamine isocyanate (TRIC) were purchased from Sigma-Aldrich (St. Louis, MO, USA). Polyclonal pig antiserum against FMDV was prepared by our laboratory. An anti-FMDV VP2 monoclonal antibody was kindly provided by Li Yu (Harbin Veterinary Research Institute, Harbin, China). Puromycin and mouse antipuromycin monoclonal antibodies were purchased from Merck Millipore (Billerica, MA, USA). Lipofectamine RNAiMAX and Lipofectamine 2000 were purchased from Invitrogen (California, USA). Mizoribine, MG132, CQ, Z-VAD-FMK, CHX, GSK-3008348, and T-1105 were purchased from MedChemExpress (Monmouth Junction, NJ). All drugs in this study were dissolved in 0.1% DMSO.

### RNAi.

For RNA interference (RNAi), siRNAs targeting candidate genes and negative-control (NC) siRNA were synthesized by Genepharma (Shanghai, China). The sequences of the siRNAs for Sus scrofa HSP60 are as follows: siRNA 1, 5′-GCAGAUGCUCGAGCCUUAATT; siRNA 2, 5′-CCAGCCUUGGAGUCAAUAATT; and siRNA 3, 5′-GCAUCAUUGAUCCAACUAATT. The sequences of the siRNAs for Sus scrofa HSP10 are as follows: siRNA 1, 5′-GGACAAGCAUUUAGAAAGUTT; siRNA 2, 5′-GAGGCACCAAAGUAGUUCUTT; and siRNA 3, 5′-GAGGCAUUAUGCUUCCAGATT.

Cells grown to 80% confluence were transfected with siRNA using Lipofectamine RNAiMAX, according to the manufacturer's instructions.

### Plasmid constructs.

Mammalian expression plasmids for the FMDV structural proteins VP0, VP1-2, and VP3 and nonstructural protein 2C and 3A and the dual-luciferase plasmid psiCHECK-FMDV were previously synthesized by our laboratory (59, 60). HA-tagged HSP60 was synthesized by GenScript (Suzhou, China). FLAG-tagged truncated 2C and 3A and GFP-tagged truncated HSP60 were synthesized by Sangon (Shanghai, China). All DNA constructs were verified by sequencing.

### Construction of rFMDV-GFP.

The subgenomic FMDV-GFP replicon (rFMDV-GFP) was constructed by replacing the Lb and P1 genes of the full-length infectious clone of FMDV O/HN/CHA/93 (pOFS-K1234) with a GFP reporter gene (61). The replicon linearized with NotI was transfected into BSR-T7 cells using Lipofectamine 2000 (Invitrogen).

### Cell viability assay.

After cells were grown to 80% confluence in 96-well plates, they were incubated with indicated drugs for 24 h or transfected with specific siRNAs or specific plasmids for 48 or 24 h, respectively. To determine the viability of indicated cells, an MTT assay was performed: 10 μL of CellTiter 96 AQueous One solution cell proliferation assay reagent (Promega, WI, USA) was directly added to the cells and incubated for 4 h. The absorbance at 490 nm was recorded with a Synergy H1 microplate reader (Biotek, USA).

### RT-qPCR.

RNAi Plus (TaKaRa) was used to extract RNA from cells, followed by reverse transcription to synthesize cDNA using 5× RT master mix (TaKaRa). HSP60, FMDV, and GAPDH transcript levels were quantified through quantitative real-time PCR (RT-qPCR). The primers specific for each gene were as follows: FMDV, 5′-CAAACCTGTGATGGCTTCGA-3′ and 5′-CCGGTACTCGTCAGGTCCA-3′; Sus scrofa GAPDH, 5′-ACATGGCCTCCAAG-GAGTAAGA-3′ and 5′-GATCGAGTTGGGGCTGTGACT-3′.

### Virus titration.

Virus infectivity was titrated by endpoint dilution. Serially diluted samples were used to infect indicated cells in 96-well plates, and the TCID_50_ was calculated using the Reed-Muench method.

### Immunoprecipitation assay.

PK-15 cells were lysed with radioimmunoprecipitation assay (RIPA) lysis buffer (Beyotime Biotechnology, Shanghai) for 1 h on ice and then centrifuged at 15,000 × *g* for 20 min at 4°C. Supernatants were immunoprecipitated with the appropriate antibodies at 4°C overnight. The immune complexes were incubated with protein G-agarose beads (GE Healthcare, Chicago, IL, USA) for 2 h, washed six times with lysis buffer, and eluted in 1× SDS-PAGE sample buffer for Western blotting.

### Ribopuromycilation assay.

PK-15 cells were infected with FMDV at an MOI of 25 in the presence of DMSO or mizoribine and then labeled with 1 μM puromycin for 1 h. After puromycin labeling, cells were washed three times at different time points using cold PBS and lysed in 1× SDS loading buffer. Equivalent amounts of protein were separated by 12% SDS-PAGE and then subjected to Western blot analysis using antipuromycin or indicated antibodies.

### SDG sedimentation.

BHK-21 cells seeded in one 175-cm^2^ cell culture flask at 80% confluence were infected with FMDV O at an MOI of 10. After 1 h adsorption, the unbound virus was washed away, and an infection medium containing DMSO or 100 μM mizoribine was added 8 hpi. Cells were collected with trypsin, washed twice with PBS, and lysed in 500 μL of TNE buffer (10 mM Tris-HCl [pH 7.4], 100 mM NaCl, 1 mM EDTA, 0.5% NP-40, 1× protease inhibitor stock) for 20 min on ice. Cellular debris was removed by low-speed centrifugation. For separation of 5S/12S and larger viral assemblies, the above-resulting supernatant was fractionated through a 5-to-25% (wt/vol) or 15-to-45% (wt/vol) sucrose density gradient, respectively, with centrifugation for 3.5 h at 35,000 × *g* in an SW40 Ti rotor (Beckman) at 4°C (zero breaks). Fractions (500 μL) were collected from the top of the gradient, and 10 μL of each odd fraction was subjected to Western blotting.

### Dual-luciferase reporter assay.

For overexpression assays, PK-15 cells were cultured in 24-well plates. When the cells reached 80% confluence, they were cotransfected with HSP60-HA and psiCHECK-FMDV using Lipofectamine 2000. Transfected cells were incubated for 24 h, and samples were harvested with lysis buffer. For knockdown assays, cells were transfected with siRNA targeting HSP60 using RNAiMAX and incubated for 30 h. According to the manufacturer's instructions, firefly and *Renilla* luciferase activities were analyzed using a dual-luciferase reporter assay system (Promega). The ratio of *FLuc* expression to *RLuc* expression shows the relative FMDV-IRES activity.

### WB.

Cells were lysed to obtain the total protein fraction. Proteins were denatured with 1× SDS loading buffer, separated by SDS-PAGE, and transferred to NC membranes. Membranes were blocked for 1 h in 5% skim milk, incubated overnight with primary antibodies, washed with Tris-buffered saline–Tween (TBST) six times, and incubated with horseradish peroxidase-conjugated secondary antibodies for 1 h again washed six times with TBST. Finally, the membranes were incubated with an enhanced chemiluminescence detection reagent (Thermo Fisher Scientific, Inc., Rockford, IL, USA) to visualize protein bands.

### Immunofluorescence assay.

To observe the colocalization of HSP60 and virus or viral proteins, PK-15 cells grown on coverslips were infected with FMDV at an MOI of 5. The indicated plasmids were cotransfected into PK15 cells for 24 h. At specific times after infection or transfection, the cells were fixed with 4% paraformaldehyde for 30 min. After three washes with PBS, the fixed cells were permeabilized with 0.1% Triton X-100 in PBS for 15 min, washed with PBS, and blocked in 5% bovine serum albumin in PBS for 1 h. The cells were then incubated with a pig anti-FMDV polyclonal antibody or an anti-HSP60, anti-HA, or anti-DDK mouse monoclonal antibody for 1 h and then with a TRIC-conjugated rabbit anti-pig IgG secondary antibody or a FITC-conjugated or TRIC-conjugated goat anti-mouse IgG secondary antibody for 1 h. The nuclei were stained with DAPI (Beyotime) for 15 min and visualized. Images were captured with a laser-scanning confocal microscope (Leica SP8; Leica, Solms, Germany).

### Transmission electron microscopy.

PK-15 cells were infected with FMDV at an MOI of 5. After 4 h postinfection, cells were fixed in a solution that contained 3% glutaraldehyde and 2% paraformaldehyde in 0.1 M cacodylate buffer (pH 7.4) for 2 h at 4°C. Cells were washed and postfixed in 1% osmium tetroxide for 1 h and then incubated in 4% uranyl acetate for 2 h at room temperature. Samples were dehydrated at 4°C in alcohol. After treatment with alcohol-Epon (1:1) for 7 h at room temperature, samples were embedded in 100% Epon resin. Polymerization of the samples was performed in an oven at 35°C for 6 h, 45°C for 6 h, and 60°C for 24 h. The embedded samples were sliced into 80-nm (65-nm) sections and poststained with 4% uranyl acetate in H_2_O and lead citrate. Images were obtained using a Hitachi HT7800 transmission electron microscope.

### Production of adenovirus expressing pAdM-shHSP60.

Three shRNAs targeting the mouse HSP60 mRNA and a nontargeting control shRNA (shNC) were designed and synthesized by Hanbio Biotechnology (Shanghai, China). Three sequences targeting HSP60 mRNA were as follows: 5′-GATCCGTGCCACTGTTCTGGCACGATCTATTCTCGAGAATAGATCGTGCCAGAACAGTGGCATTTTTTG-3′, 5′-GATCCGATGTTGGCTGTGGATGCTGTAATTCTCGAGAATTACAGCATCCACAGCCAACATCTTTTTTG-3′, and 5′-GATCCGTCAGTCCATTGTCCCTGCTCTTGAACTCGAGTTCAAGAGCAGGGACAATGGACTGATTTTTTG-3′. These sequences were cloned into the vector pHBLV-shRNA-ZsGreen-PURO, and adenoviruses carrying the recombinant vector were generated as previously described (62).

### Viral challenge in suckling mice.

Groups of 2-day-old BALB/c mice (suckling mice; *n* = 10 per group) were purchased from the Laboratory Animal Center of Lanzhou Veterinary Research Institute, Chinese Academy of Agricultural Sciences (Lanzhou, China). The LD_50_ of FMDV strain O/China/99 for suckling mice was estimated by the Reed-Muench method. The suckling mice were subcutaneously inoculated in the neck with 1.0 × 10^8^ PFU of purified pAdM-shNC or pAdM-shHSP60 in 100 mL of PBS. At 72 h after inoculation, the mice were challenged with a subcutaneous injection of 20 LD_50_ of O/China/99 in 100 mL of PBS at the inoculation sites. The survival rate of the suckling mice was monitored for 7 days after the challenge.

### Statistical analysis.

The data presented in this paper are expressed as means and standard deviations (SD) for at least two replicates and were evaluated with a two-tailed Student's *t* test or two-way analysis of variance (ANOVA) in GraphPad Prism software version 9 (GraphPad, La Jolla, CA, USA).
