# Supplementary figures and images for: Heat Shock Protein 60 Is Involved in Viral Replication Complex Formation and Facilitates Foot and Mouth Virus Replication by Stabilizing Viral Nonstructural Proteins 3A and 2C
Source: mBio. 2022 Sep 15;13(5):e01434-22. doi: 10.1128/mbio.01434-22 (PMC9601101; doi:10.1128/mbio.01434-22)

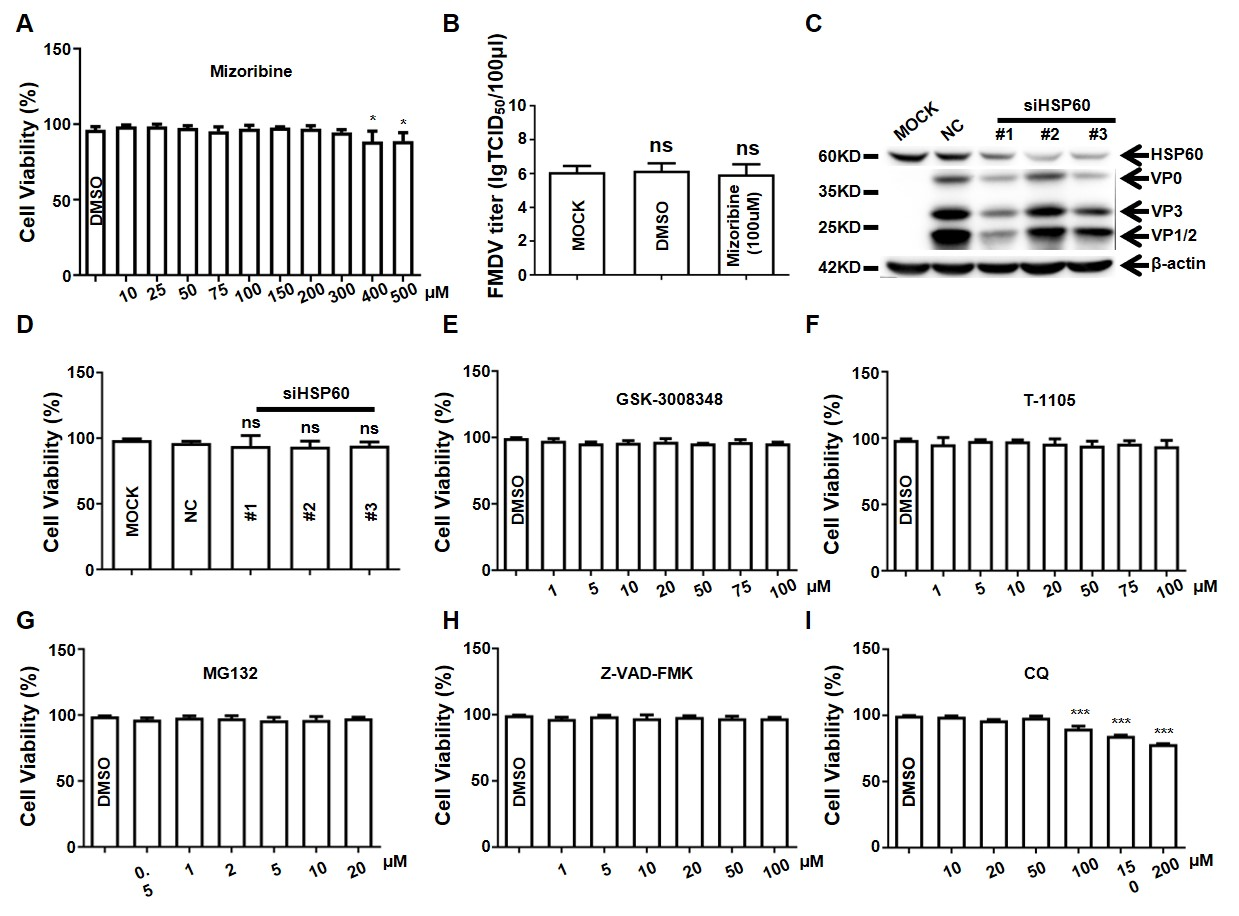

Supplement: FIG S1 [file mbio.01434-22-s0001.tif]

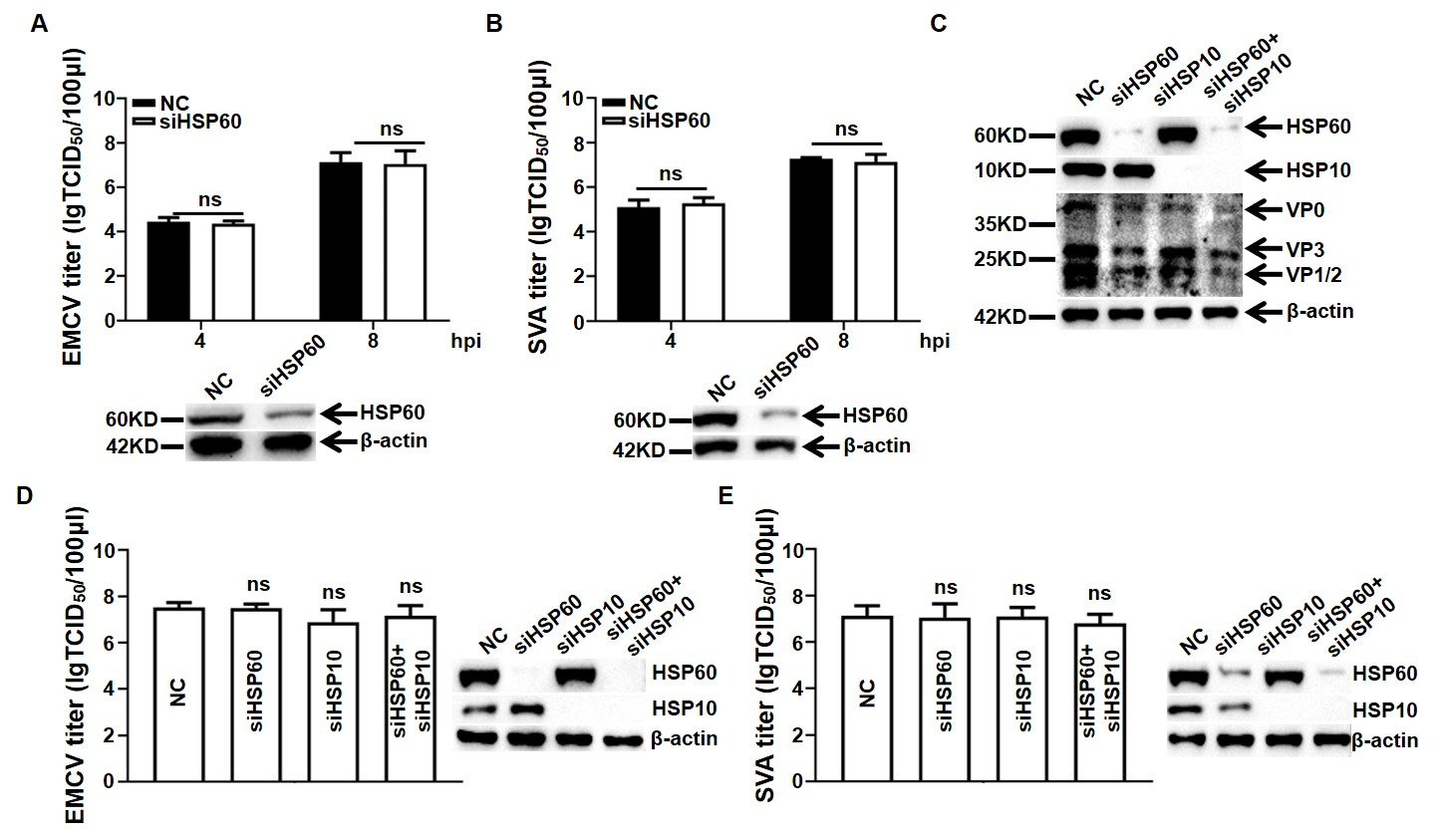

Supplement: FIG S2 [file mbio.01434-22-s0002.tif]

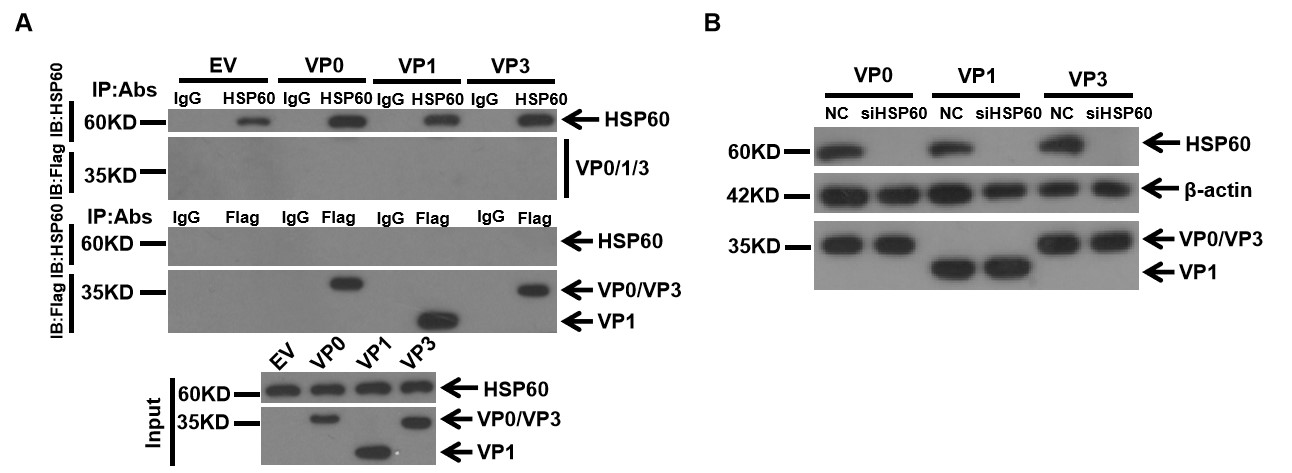

Supplement: FIG S3 [file mbio.01434-22-s0003.tif]

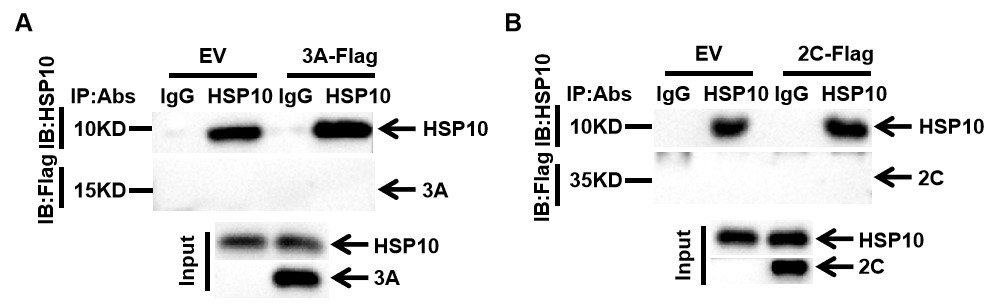

Supplement: FIG S4 [file mbio.01434-22-s0004.tif]
